# Emergent Patterns of Social Affiliation in Primates, a Model

**DOI:** 10.1371/journal.pcbi.1000630

**Published:** 2009-12-24

**Authors:** Ivan Puga-Gonzalez, Hanno Hildenbrandt, Charlotte K. Hemelrijk

**Affiliations:** Theoretical Biology, Centre for Ecological and Evolutionary Studies, University of Groningen, Groningen, The Netherlands; University College London, United Kingdom

## Abstract

Many patterns of affiliative behaviour have been described for primates, for instance: reciprocation and exchange of grooming, grooming others of similar rank, reconciliation of fights, and preferential reconciliation with more valuable partners. For these patterns several functions and underlying cognitive processes have been suggested. It is, however, difficult to imagine how animals may combine these diverse considerations in their mind. Although the co-variation hypothesis, by limiting the social possibilities an individual has, constrains the number of cognitive considerations an individual has to take, it does not present an integrated theory of affiliative patterns either. In the present paper, after surveying patterns of affiliation in egalitarian and despotic macaques, we use an individual-based model with a high potential for self-organisation as a starting point for such an integrative approach. In our model, called GrooFiWorld, individuals group and, upon meeting each other, may perform a dominance interaction of which the outcomes of winning and losing are self-reinforcing. Besides, if individuals think they will be defeated, they consider grooming others. Here, the greater their anxiety is, the greater their “motivation” to groom others. Our model generates patterns similar to many affiliative patterns of empirical data. By merely increasing the intensity of aggression, affiliative patterns in the model change from those resembling egalitarian macaques to those resembling despotic ones. Our model produces such patterns without assuming in the mind of the individual the specific cognitive processes that are usually thought to underlie these patterns (such as recordkeeping of the acts given and received, a tendency to exchange, memory of the former fight, selective attraction to the former opponent, and estimation of the value of a relationship). Our model can be used as a null model to increase our understanding of affiliative behaviour among primates, in particular macaques.

## Author Summary

Individual primates distribute their affiliative behaviour (such as grooming) in complex patterns among their group members. For instance, they reciprocate grooming, direct it more to partners the higher the partner's rank, use it to reconcile fights and do so in particular with partners that are more valuable. For several types of patterns (such as reconciliation and exchange), a separate theory based on specific cognitive processes has been developed (such as individual recordkeeping, a tendency to exchange, selective attraction to the former opponent, and estimation of the value of a relationship). It is difficult to imagine how these separate theories can all be integrated scientifically and how these processes can be combined in the animal's mind. To solve this problem, we first surveyed the empirical patterns and then we developed an individual-based model (called GrooFiWorld) in which individuals group, compete and groom. The grooming rule is based on grooming out of fear of defeat and on the anxiety reducing effects of grooming. We show that in this context this rule alone can explain many of the patterns of affiliation as well as the differences between egalitarian and despotic species. Our model can be used as a null model to increase our understanding of affiliative patterns of primates.

## Introduction

Patterns of affiliative behaviour have long puzzled primatologists. One of the most frequent behavioural acts is grooming. It has been explained as serving several functions, such as cleaning the fur [1], reducing anxiety, tension and stress [2], social bonding [3], repairing relationships [4] and social reciprocation and exchange [5]. As regards the mechanisms of exchange, individuals have been supposed to direct grooming up the hierarchy in order to receive more effective support in return, and due to competition for partners of high rank they may end up grooming others of similar rank [6]. Besides, they were also supposed to groom others of similar rank, because individuals of similar rank have similar needs [7]. Grooming between two former opponents immediately after a fight has been interpreted to function as a means to repair the relationship or ‘reconcile’, because it occurred significantly earlier after a fight than otherwise in matching control periods the next day. Besides, individuals appeared to reconcile in particular with those partners that appeared more valuable to them, the so-called ‘valuable-relationship hypothesis’ [8].

To complicate matters, the degree of exchange and reciprocation [9] appeared to differ between egalitarian and despotic species. Applying market theory [10],[11], this was explained by assuming that the exchange rate of services differed between the two competitive regimes [9]. Further, the co-variation hypothesis explained the lower conciliatory tendency in despotic societies by the greater danger involved in reconciliation in these species [12].

Many specific cognitive considerations have been suggested to underlie these affiliative patterns. For instance, as regards reciprocity and exchange, the individuals are supposed to keep records of the acts of grooming and tune them to frequencies of receipt of being groomed or another act, such as support [13], and to use their knowledge of the ranks of others to obtain more effective support [6],[14]. Besides, individuals have been supposed to be attracted to others of higher rank [6] and to others of similar rank [7]. The supposed cognition underlying reconciliation consists of the ability to remember the former opponent and of selective attraction to the former opponent or a conciliatory disposition [15],[16]. As to their inclination particularly to reconcile fights with opponents that are of greater value to them, the so-called ‘valuable-relationship hypothesis’ [8], [17]–[23], three key components are supposed to influence the quality of a relationship, namely its security, its value, and the compatibility of both partners [8],[24]. According to Silk [25] this implies that assessing the value of a relationship over the long-term requires cognitive sophistication, because it asks for a precise memory of what happened in the past and for a correct evaluation of the relationship in the long run.

These theories of affiliation pose several problems. First, evidence for each of these theories is not conclusive [5], [26]–[30]. Second, from a scientific perspective, these numerous different theories for specific patterns of affiliation (such as exchange and reconciliation) must be integrated in some way. Third, the use of grooming as a ‘currency of exchange’ is dangerously anthropomorphic according to us and others [25],[31],[32]. As a more parsimonious alternative, we suggest to follow a more distributed approach based on local interactions and rules of thumb [31], [33]–[36]. Fourth, even though primates are obviously intelligent [37],[38] it seems much to ask of primates to combine intentionally all these rational considerations in the distribution of their affiliative behaviour (e.g. to consider what incidence of grooming was used in exchange for something, and what for reconciliation or maintenance and development of social bonds). Fifth, often simple rules suffice to cause many of the observed patterns and herewith an integrative theory [39],[40]. Therefore fewer cognitive processes may suffice as shown for instance in a model for dominance style [32],[41]. A similar integrative approach based on fewer cognitive processes is also suggested by the co-variation hypothesis (or theory of social epigenesis). In this theory part of the behavioural acts is supposed to be forced by constraints due to the specifics of the social structure [12].

For these reasons, we use in the present paper a computer model to develop an integrative approach to patterns of social affiliation in primates. We first precede this by a survey of the precise patterns of dominance style and affiliation found in the literature. In the model, we assume very little cognitive deliberations by the individuals to groom others: Individuals merely groom others out of fear of being defeated and to reduce their own anxiety. Individuals do not intend to reconcile fights nor to exchange or reciprocate grooming. Our model is an extension of our earlier model of grouping and competition, called DomWorld [42],[43]. We choose DomWorld, because it has reproduced many of the patterns of aggression, dominance and spatial structure that have been observed in despotic and egalitarian societies of primates, in particular of macaques. These have arisen merely as a side effect of local rules for grouping and competition through the feedback between hierarchical development and spatial-social structure with dominants in the centre and subordinates at the periphery [35], [41], [44]–[46]. Note that the hierarchy develops via self-reinforcing effects of victory and defeat, which have been described for many species including primates [45], [47]–[50]. Through these self-reinforcing effects, occasional victories of low ranking individuals may lead to rank reversals. This is important, because dominance hierarchies in empirical data are not entirely stable [51]–[55].

Interactions in our new model, called GrooFiWorld (a contraction of groom and fight), are extended with the option to groom. When individuals meet each other at close proximity, they will consider whether to groom, to fight or to rest. As to the order of what to do first, we are led by four observations: first, those on baboons by Kummer [56] who inform us that upon their first encounter individuals first fight and later groom; second, by the empirical finding that an individual builds up anxiety (as indicated by the increased heart rate) when approaching an opponent by whom it may be defeated [rhesus monkeys, 57]; third, that anxiety increases after a fight as is indicated by the increase in frequency of scratching and heart rate in both opponents [25], [58]–[64]; fourth, that anxiety may subsequently be reduced (in many species) by the receipt of affiliative behaviour as indicated by the reduced heart rate and the rate of self-directed behaviour [57],[61],[62],[64] and to a lesser degree by active grooming [65]. Furthermore, our model is informed by empirical studies on grooming and opiate administration which indicate that not being groomed for some length of time reduces the concentration of endorphins and increases the motivation to be groomed, and that grooming increases the level of endorphins in the brain and reduces the motivation to groom [66]–[71].

In sum upon encountering someone else, an individual in our model first deliberates whether or not to attack. This decision depends on the risks involved (whereby risk concerns the chance of losing a fight), as is the case among primates [72], and as in our earlier model: a fight is only initiated when the individual expects to win [41],[73]. If defeat is expected, its fear of losing makes the individual consider grooming the other. Its decision whether or not to groom depends on its degree of anxiety: an individual that is more anxious is more inclined to groom (instead of resting close by). After being groomed by another and (a little less) after actively grooming another, its anxiety and therefore its tendency to groom diminishes. Its anxiety also increases after a fight and after a period of not having been involved in grooming. Note that we do not distinguish between anxiety, tension or stress.

In order to compare the patterns of affiliative behaviour in our model with those in real primates, we used the same statistical measures as applied in empirical data and we confined ourselves to macaques for two reasons. First, because their social behaviour has been studied extensively and shown to differ in interesting ways between the typical egalitarian and despotic societies [74],[75]. Second, because in our earlier model, DomWorld patterns of dominance and aggressive interaction were remarkably similar to those of macaques [41],[45]. Since GrooFiWorld is an extension of this model, we assume it to also be suitable for comparing to macaques.

The paper is organized as follows. First, we summarise the literature on the common patterns of affiliative behaviour in females of egalitarian and despotic species of macaques (Table 1). Second, we tune the percentage of grooming time and the unexpectedly emerging percentage of reconciliation to empirical data for despotic societies. Third, by varying the intensity of aggression we show the emergence of all these common patterns of affiliation and their differences between typical egalitarian and despotic macaque species in GrooFiWorld. Fourth, in order to understand how these patterns emerge, we remove different assumptions in turn, such as the self-reinforcing effects of victory and defeat and effects of spatial proximity. Fifth, the explanation of the causation of these patterns in the model leads to new hypotheses about the interconnection between other traits which we confirm in the model. Part of these predicted patterns appear also to be found in empirical data described by scientists in other contexts. Other patterns still need to be tested empirically. Since for all patterns empirical data are insufficient, we list them together in Table 2 so that the relevance of our model to empirical data may be tested in the future.

**Table 1:** Dominance style and affiliative patterns for different species of macaques, D = despotic, E = egalitarian.

Species	*Macaca mulatta*	*Macaca fuscata*	*Macaca assamensis*	*Macaca thibetana*	*Macaca nemestrina*	*Macaca fascicularis*	*Macaca sylvanus*	*Macaca radiata*	*Macaca arctoides*	*Macaca silenus*	*Macaca nigra*	*Macaca tonkeana*
Dominance style	D^1^	D^1^	D^1^	D^2^	D^3^	D^1^	E^1^	E^1^	E^1^	E^1^ *	E^1^	E^1^
Unidirectionality aggression^1^	True	True	True	True	True	True	Not true	Not true	Not true	Not true	Not true	Not true
Frequency of aggression	Low^5^	NA	NA	NA	NA	NA	NA	NA	High^5^	NA	NA	High^6^
Interindividual distance	High^5^	NA	NA	NA	NA	NA	NA	NA	Low^5^	NA	NA	Low^6^
Centrality of dominants	NA	True^4^	NA	NA	NA	NA	NA	NA	NA	NA	NA	NA
Conciliatory tendency	∼7%^1^	∼10%^1^	∼11%^7^	∼6%^8^	∼32%^9^	∼18%^1^	∼20%^1^	∼29%^10^	∼35%^11^	∼70%^1^	∼50%^1^	∼50%^1^
Grooming reciprocation	True^12^	True^12^	NA	NA	NA	True^12^	True^12^	True^12^	True^12^	NA	NA	True^6^
Grooming up the hierarchy	True^13^^,^ 14	True^13^^,^ 15 ^,^ 16	True^17^	NA	NA	True^13^^,^ 14	NA	NA	Not true^18^	NA	NA	Not true^6^
Grooming partners of similar rank	True^13^^,^ 14	True^13^^,^ 16	NA	NA	NA	True^13^^,^ 14	NA	NA	Not true^18^	NA	NA	Not true^6^^,^ 18
Reconciliation with valuable partners	NA	True^19^	True^20^	True^8^	True^9^	True^21^	NA	NA	True^11^	NA	NA	NA

*: indicates that there is debate about the classification of the dominance style of this species.

1 [12].

2 [51].

3 [105].

4 [107]–[109].

5 [106],[150].

6 [80],[118].

7 [112].

8 [151].

9 [18],[152].

10 [63].

11 [19],[29],[153].

12 [154].

13 [155].

14 [7].

15 [27].

16 [156].

17 [105].

18 [157].

19 [21],[125].

20 [20].

21 [158].

**Table 2:** List of model based hypotheses that emerge in the model.

Model-based hypotheses	Empirical Data
**A) In general:**	
1) Positive correlation between proximity and grooming	[52],[106],[118]
2) No correlation between frequency of grooming by an individual and its rank	pro: [128],[129] contra: [52].
3) Positive correlation between grooming up the hierarchy and the gradient of the hierarchy	[115]
4) Positive association between grooming others of similar rank and spatial centrality of dominants	[7]
5) Positive correlation between % time grooming and % reconciliation in group	Not available
6) Positive correlation between % interactions spent in grooming and % reconciliation in group	Not available
7) Negative association between spatial rigidity and conciliatory tendency	Not available
**B) In despotic species:**	
1) Conciliatory tendency directed up the hierarchy	[127]
**C) In despotic species (compared to egalitarian ones):**	
1) The gradient of the hierarchy is steeper	[9],[159],[160]
2) Higher ranking individuals are more often aggressive	Not available
3) Higher ranking individuals receive less aggression	Not available
4) Lower ranking individuals lose more fights	Not available
5) Percentage of fighting is lower	[106]
6) Distance among group members is larger	[103],[106],[116],[117]
7) The spatial structure (with dominants in the centre) is stronger	Not available
8) The time spent grooming is lower	[106]
9) Percentage of interactions spent in grooming is lower	Not available
10) The diversity of neighbours is lower	Not available
11) Stronger association between spatial proximity of partner and conciliatory tendency	Not available
12) Negative correlation between dominance and anxiety is stronger	Not available
13) The percentage with which females groom males is lower	Not available

## Methods

### The model

A demo of our model can be seen in Video S1. The model is individual-oriented and event-driven [76]. It has been written in C++, as an extension of a previous model of grouping and competition, called *Dom*-World [41],[42],[77],[78] which has been reimplemented in C++ by Hanno Hildenbrandt. The extension consists of the addition of grooming behaviour (for default parameters see Table 3). Therefore, we call it ‘GrooFiWorld’. The individuals are provided with three tendencies: 1) to group, 2) to perform dominance interactions and 3) to display affiliative behaviour. Why individuals actually group (whether to avoid predators or because resources are clumped) is not specified and irrelevant to the model. The same holds for dominance interactions which may reflect competition for resources such as food and mates, but these resources are not explicitly specified in the model. Individuals groom to reduce *Anxiety*, as suggested for real primates [66]–[71], [79]–[81].

**Table 3:** Default parameter values in ‘GrooFiWorld’.

Parameter	Description	Females	Males
**General Parameters**			
Total Individuals	Total number of individuals	12	
Sex ratio (at high aggression intensity)	Number of males and females	10	2
Sex ratio (at low aggression intensity)	Number of males and females	8	4
InitRadius	Predefined space at start of simulation	1.7*# Inds	1.7*# Inds
**Grouping Parameters**			
Perspace	Close encounter distance	8	8
Nearview	Medium distance	24	24
MaxView	Maximal viewing distance	50	50
SearchAngle	Turning angle to find others	90°	90°
VisionAngle	Angle of field of view	120°	120°
**Dominance Parameters**			
InitDom	Initial Dom value	16	32
RiskSens	Number of ‘mental battles’	2	2
StepDom (high aggression intensity)	Scaling factor for aggression intensity	0.8	1
StepDom(low aggression intensity)	Scaling factor for aggression intensity	0.08	0.1
Fleeing Dist	After loosing a fight	2	2
**Grooming Parameters**			
InitAnx	Initial anxiety value	0.5	0.5
AnxInc	Increase in anxiety after every activation	1%	1%
AnxDcrGree	Decrease in anxiety in groomee	0.15	0.15
AnxDcrGrmr	Decrease in anxiety in groomer	0.1	0.1
AnxIncFight	Increase in anxiety after fighting	0.1	0.1

GrooFiWorld consists of a ‘world’ (without borders) containing its interacting individuals. The space of the ‘world’ is continuous, i.e. individuals are free to move in any direction. Individuals have a certain angle of vision (*VisionAngle*) and a maximum distance of perception (*MaxView*). At the start of each run they occupy random locations within a predefined circumference, *InitRadius*, which is the product of an arbitrary constant times the number of individuals.

Activities of individuals are regulated by a timing regime in which each individual receives a random waiting time from a uniform distribution and the individual with the shortest waiting time is activated first. This regime is combined with a biologically plausible timing regime reflecting a kind of social facilitation [e.g., see 82] in which the waiting time of an individual is shortened when a dominance interaction occurs close by (within the individual's *NearView*).

### Grouping rules

**Figure 1:**
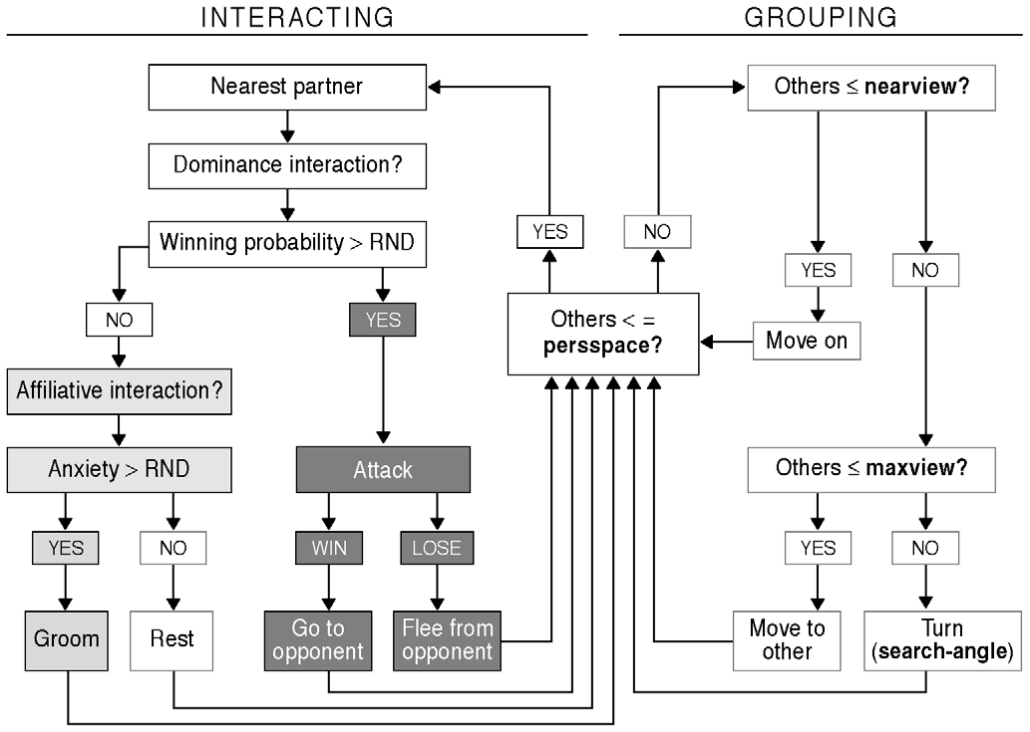
Rules of behavioural interaction. In light grey boxes the new rules of GrooFiWorld related to grooming are indicated. In white boxes the grouping rules, and in black boxes the rules for dominance interactions from DomWorld [41],[43].

Whenever an individual does not see another one close by (within its personal space, *PersSpace*), grouping rules come into effect. The individual starts looking for others at greater and greater distances (*NearView* and *MaxView*). If, even then, no one else is in sight, it turns over a *SearchAngle* in order to scan for others. In this way individuals tend to remain in a group (Figure 1 and Table 3).

If, however, an individual spots another one close by, within its personal space (*PersSpace*), a social interaction may take place.

### Interactions

Upon encountering someone else the individual first deliberates whether or not it will perform a dominance interaction on the basis of the risk of losing the fight [following the so–called ‘risk–sensitive attack strategy’, 43]. Only if it expects to be defeated, it will consider grooming. In real primates, motivation to groom depends on opiate concentrations as well as on other physiological conditions such as stress levels, and we have coded these factors together as *Anxiety* [66]–[68],[71],[83] (Figure 1). Thus, in GrooFiWorld, first, the more anxious an individual is the more likely it is to groom (instead of resting close by); second, after being groomed and (a little less) after actively grooming another, an individual's anxiety and thus its tendency to groom declines; third, after not having been involved in grooming for some time an individual's anxiety builds up again; and fourth, an individual's anxiety grows after a fight. Thus anxiety reflects the psychological and physiological state of an individual.

### Dominance rules

Dominance interactions are modelled as before [41],[46] and they are an extension of the DoDom rules of Hogeweg [46]. First, an individual *i* estimates whether it will win on the basis of a ‘mental battle’ (Equation 1). It may do so once [84] or repeatedly depending on its degree of sensitivity to risks (*RiskSens* Table 3 and Parameters and Experimental Setup). Higher values of *RiskSens* indicate that individuals need to win several mental fights before starting an actual interaction. Here, individuals *i* and *j* observe each other's capacity of winning, i.e. their dominance values *Dom*_i_ and *Dom*_j_. The probability of winning for individual *i* is greater if it is higher in rank, and this is proportional to the *Dom*-value of individual *i* relative to that of its opponent *j* (Equation 1). It expects to be victorious if its relative dominance value is greater than a random value drawn from a uniform distribution between zero and one. If this is the case, a dominance interaction takes place. During the actual dominance interaction, the individual *i* compares its relative dominance value again with another value randomly drawn and if its relative dominance value is greater than a new random number, it wins (w_i_ = 1), else it loses (w_i_ = 0):
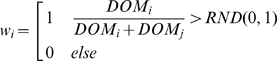
(1)The stochastic effect is introduced to allow for dominance reversals. To reflect the self-reinforcing effects of victory and defeat [45],[85], dominance values are updated by increasing the dominance value of the winner and decreasing that of the loser by the same amount:
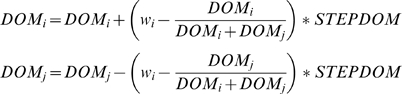
(2)


This positive feedback is ‘dampened’ because a victory of a higher ranking opponent increases its relative *Dom*-value only slightly, whereas an (unexpected) success of the lower ranking individual increases its relative dominance value by a greater change. To keep *Dom*-values positive, their minimum value is, arbitrarily, set at 0.01.

The change in *Dom*-values is multiplied by a scaling factor, called *StepDom*, which varies between 0 and 1 and represents the intensity of aggression [41],[84] (see Parameters and Experimental setup). High values imply a great change in *Dom*-value after a fight, and thus indicate that single interactions (e.g. involving biting) may strongly influence the future outcome of conflicts. Conversely, low *StepDom*-values represent low impact (e.g. threats or slaps).

Winning an interaction includes chasing the opponent over a distance of one unit and then turning randomly 45 degrees to right or left in order to reduce the chance of repeated interactions between the same opponents. The loser responds by fleeing under a small random angle over a predefined *FleeingDistance*.

### Grooming rules

If an individual meets another in its *PersSpace* and when it has decided on the basis of a ‘mental’ battle that it is too dangerous to attack, the individual considers whether or not to groom its partner (Figure 1). Grooming behaviour is induced by the level of *Anxiety*, which ranges from very relaxed to very tense, represented by a scale from 0 to 1. If the *Anxiety* value is lower than a random number, the individual will display ‘non-aggressive’ proximity; otherwise, if *Anxiety* is higher, it will groom its partner (Figure 1). After grooming both partners turn over a small angle (45°) randomly to the right or left in order to avoid repeated interactions with the same partner. Grooming reduces *Anxiety*. In line with empirical evidence [61], [62], [64]–[71], it does so more strongly in the groomee (with *AnxDcrGree*) than in the groomer (with *AnxDcrGrmr*) (Table 3). During periods without grooming *Anxiety* increases, which is consistent with opiate-dependent motivation to groom in real primates [67],[71]. This increase is updated after every activation with *AnxInc*. Furthermore, inspired by the observed increase in scratching after a fight in real primates [8], in the model, after a fight *Anxiety* increases with *AnxIncFght* for both opponents.

### Parameters and experimental set-up

Many parameters that have been used in earlier studies were kept at the same value, namely the *NearView*, *MaxView*, *FleeingDist*, *SearchAngle* and *StepDom* values. Note that *StepDom* values (that reflect intensity of aggression) differ between the sexes (on the basis of the stronger muscular structure of males than females) and between dominance styles reflecting the tendency of individuals in despotic societies to bite relatively more (and slap and threaten less) than in egalitarian ones [41],[42] [78] [84] [86] (Table 3). We used 12 individuals to represent the number of adults in a group of primates. Since in empirical studies the percentage of females is lower in egalitarian macaques with approximately 70% females than despotic macaques with approximately 80% females, we have set the sex ratio at low and high aggression intensity accordingly (with 8 females, 4 males at low intensity and 10 females and 2 males at high intensity) [87]–[89]. The initial dominance values we set at 16 for females and 32 for males, reflecting the initially higher winning chance of males due to sexual dimorphism in fighting power resulting from differences in body weight, physiology and weaponry.

In order to tune the frequency of grooming to 20% of the time as indicated for despotic societies of real primates by Dunbar [90], we had to increase *PerSpace* from 4 to 8 units (reflecting a tendency to interact with others over larger distances), to increase the risk-sensitivity of individuals by increasing the number of mental battles ‘ego’ had to win before starting a real dominance interaction (in order to reduce the frequency of aggression) (*RiskSens*, Table 3) and to tune the *Anxiety*-related parameters (see Table 3).

To understand what caused the patterns of affiliation in the model, each of four assumptions related to grooming and fighting were switched off in turn. The simulation was run in turn 1) without the self-reinforcing effects of winning and losing fights, 2) without the grooming inducing effect of anxiety, 3) without the dependence of grooming on the risks to attack and 4) without the selection of interaction partners on the basis of spatial proximity.

First, when switching off the self-reinforcing effects of winning and losing, we gave the individuals *Dom* values that were constant. We took these values from runs with the corresponding intensity of aggression, because hierarchical differentiation was greater at a high than at a low *StepDom*. We took the values from the middle (i.e. period 230) of the interval between periods 200 and 260, because in this interval the *Dom* values were considered to have stabilised [91], since the average *Dom* values between period 200 and 230 are significantly correlated with those between 230 and 260 (Kendall Tau, N = 10, High intensity Tau = 0.88 ***, Low intensity Tau = 0.85** two tailed probability).

Second, to switch off the grooming inducing effect of *Anxiety* implies that we made grooming independent of the value of *Anxiety*. In this case, the individual always groomed its partner whenever it refrained from attack because of high risks.

Third, switching off fear-based grooming, implied that we made grooming independent of the risks of defeat, i.e. upon meeting another individual in its *PerSpace* we gave the individual a 50% chance to either consider grooming it or attacking it. After choosing between attacking and grooming, the risk-sensitive decision procedure was used to decide whether the individual actually attacked and the anxiety-based rule was used to decide whether it actually groomed. If the individual decided to refrain from interacting, it rested at its location.

Fourth, to switch off proximity-based interactions, interaction partners were chosen at random independent of their proximity in space.

### Data collection and analysis

Every run consisted of 260 periods and each period consisted of 240 activations (the number of individuals (i.e. 12) times 20). Data were collected from period 200 to 260 to exclude any bias caused by transient values. Data consisted of every change in spatial position and in heading direction of each individual and, as regards social interactions, we recorded (1) the identity of the attacker and its opponent, (2) that of the winner/loser, (3) the updated *Dom* values of the individuals, (4) the identity of the groomer and groomee and (5) the updated *Anxiety* value of the individuals. For each model (the complete model and the four controls with a missing assumption) 10 independent simulations were run for each of the two aggression intensities (high and low). The results are shown here per condition as the average statistic of these 10 runs with their combined probability using the improved Bonferroni procedure [92]. Patterns apparent in empirical studies of egalitarian and despotic macaques (Table 1) were tested for by means of (combined) one-tailed probabilities (Tables 4 and 5), all the other patterns were tested with two-tailed probabilities (Tables 6 and 7). To test for differences in patterns between high and low intensity of aggression, we used the Mann Whitney U test whereby we compared the statistics between 10 runs at a high and 10 runs at a low intensity of aggression (see Tables 4, 5, 6).

**Table 4:** Dominance style and affiliative patterns (for measurements see methods).

	Real macaque societies		Model	
Intensity of aggression	Despotic	Egalitarian	High	Low
**Dominance Style**				
1) Gradient of Hierarchy	NA	NA	0.86	0.11
Gradient of the hierarchy High>Low	NA		U = 100 ***	
2) Unidirectionality of aggression	True	NS	−0.45**	0.18*
Unidirectionality of aggression High>Low	True		U = 99 ***	
3) Time spent fighting (%)			15–16%	16–18%
Fighting % High<Low	NA		U = 97 ***	
4) Mean distance among group members	Low	High	17	10
Average distance High<Low	NA		U = 100 ***	
5) Centrality of Dominants (Tau)	True	NA	−0.56*	0.06
Centrality High>Low	NA		U = 100 ***	
**Affiliative patterns**				
6) Time spent grooming (%)	8–15%	NA	***13–23%***	28–30%
7) Conciliatory tendency	7–18%^1^	20–50%^2^	***7–17%***	16–22%
Conciliatory tendency High<Low	True^1^		U = 98 ***	
**TauKr Correlations**				
8) Grooming Reciprocation	True	True	0.31**	0.45**
9) Grooming up the hierarchy	True	NS	0.44**	0.05
10) Grooming partners of similar rank	True	NS	0.25**	0.04
11) Reconciliation with valuable partners	True	True	0.21**	**−0.04**

One tailed p-values of tests are combined via the improved Bonferroni method (n = 10): * p<0.05, **p<0.01, ***p<0.001. In bold italics are the two percentages which quantities were tuned to empirical data (although reconciliation in itself was emergent). In bold are results that differ from empirical data.

1,2 Data of conciliatory tendencies of *Macaca nemestrina* ^1^ and *Macaca silenus* ^2^ were excluded, because these were considered as outliers.

**Table 5:** Dominance style and affiliative patterns when taking out different assumptions (see methods).

	No self-reinforcing effects		No anxiety induced grooming		No fear of defeat		No spatial structure	
Intensity of aggression	High	Low	High	Low	High	Low	High	Low
**Dominance Style**								
1) Gradient of the hierarchy	0.73	0.08	0.74	0.09	0.62	0.07	0.75	0.10
Gradient of the hierarchy High>Low	U = 100 ***		U = 100 ***		U = 100 ***		U = 100 ***	
2) Unidirectionality of aggression	−0.41**	0.16**	−0.39**	0.20**	−0.17*	0.25**	−0.68**	−0.15*
Unidirectionality of aggression High>Low	U = 100 ***		U = 100 ***		U = 97 ***		U = 100 ***	
3) Time spent fighting (%)	14–15%	17–18%	12–15%	13–14%	**7–9%**	**6–7%**	**31–37%**	**26–27%**
Fighting % High<Low	U = 100 ***		**U = 50.5 NS**		**U = 96 NS**		**U = 100 NS**	
4) Average distance among group-members	17.07	10.13	16.83	11.68	18.65	15.51	**NA**	**NA**
Average distance High<Low	U = 100 ***		U = 97 ***		U = 96 ***		**NA**	
5) Centrality of dominants	−0.52**	−0.10	−0.49*	−0.27	−0.41*	0.04	**NA**	**NA**
Centrality High>Low	U = 90 **		U = 76 *		U = 90 **		**NA**	
**Affiliative Patterns**								
6) Time spent grooming (%)	16–18%	27–30%	16–32%	**34–38%**	19–24%	22–27%	**41–42%**	**41%**
7) Conciliatory tendency	10–14%	14–21%	5–20%	19–25%	**19–28%**	20–28%	**0–2%**	**0–5%**
Conciliatory tendency High<Low	U = 100 ***		U = 99 ***		**U = 56 NS**		**U = 68 NS**	
**Tau-Kr Correlations**								
8) Grooming reciprocation	0.39**	0.50**	0.33**	0.47**	0.69***	0.67***	**−0.52**	**0.00**
9) Grooming up the hierarchy	0.46**	0.06	0.39**	0.11*	**0.01**	0.02	0.59**	0.07
10) Grooming those of similar rank	0.18*	0.06	0.18**	−0.01	**0.06**	0.04	**0.08**	−0.05
11) Reconciliation with valuable partners	0.23*	−0.03	0.17*	−0.03	0.04*	0.04	**NA**	**NA**

One tailed p-values of tests are combined via the improved Bonferroni method: * p<0.05, **p<0.01, ***p<0.001. Results that differ from the complete model (in Table 4) are shown in **bold**.

**Table 6:** Different variables correlated to the ranks of individuals for the model when taking out different assumptions (see methods).

	Model		No self-reinforcing effects		No anxiety induced grooming		No fear of defeat		No spatial structure	
	High	Low	High	Low	High	Low	High	Low	High	Low
1) Berger-Parker dominance index for grooming partners	0.24	0.20	0.22	0.20	0.21	0.20	0.22	0.23	0.19	0.18
**Correlations between rank and** 1										
2) Aggression given	0.84**	0.61*	0.82**	0.56	0.83**	0.53*	0.74**	0.33	0.89***	0.58*
3) Aggression received	−0.86***	−0.52*	−0.90***	−0.54	−0.89**	−0.38	−0.86***	−0.40	−0.90***	−0.61*
4) Number of fights lost	−0.86**	−0.51	−0.84**	−0.39	−0.85**	−0.44	−0.78**	−0.45	−0.87***	−0.57**
5) Anxiety	−0.59**	−0.17	−0.61*	−0.09	−0.43	−0.25	−0.25*	0.00	−0.54*	0.18
6) Grooming given	0.02	−0.14	0.20	−0.09	−0.21	0.00	0.52*	0.04	−0.85**	−0.48*
**Correlations between** 2										
7) Conciliatory tendency and proximity of the partner	−0.16	0.04	−0.19	−0.01	−0.12	0.05	0.02	−0.07*	NA	NA
8) Grooming and proximity of the partner	−0.55**	−0.42**	−0.56**	−0.42**	−0.50**	−0.40**	−0.42**	−0.46**	NA	NA
9) Conciliatory tendency and rank of the partner	0.21**	−0.01	0.25*	0.01	0.10	−0.05	−0.14	−0.04	NA	NA
**Difference between aggression intensities for** 3										
10) Aggression given	U = 88 **		U = 92 **		U = 91 **		U = 90 **		U = 88 **	
11) Aggression received	U = 89 **		U = 98 ***		U = 100 ***		U = 100 ***		U = 96 ***	
12) Number of fights lost	U = 98 ***		U = 99 ***		U = 97 ***		U = 90 **		U = 86 **	
13) Grooming-time percentage	U = 100 ***		U = 100 ***		U = 100 ***		U = 88 **		**U = 56 NS**	
14) Grooming-interaction percentage	U = 100***		U = 100***		U = 100***		U = 99**		U = 100***	
15) Grooming reciprocation (Tau-Kr)	U = 68 NS		U = 78 *		U = 81 *		U = 66NS		**U = 100** ***	
16) Berger-Parker dominance index	U = 87**		U = 100 ***		U = 61NS		U = 58NS		U = 74NS	
17) Rank and anxiety (tau)	U = 96 ***		U = 91 **		U = 68 NS		U = 110 NS		U = 67 ***	
18) Grooming up the hierarchy	U = 100***		U = 100 ***		U = 100 ***		U = 55 NS		U = 100 ***	
19) Grooming those of similar rank	U = 95 ***		U = 70 NS		U = 85 **		U = 51 NS		U = 84 **	
20) Grooming and proximity of the partner (Tau-Kr)	U = 77 *		U = 90 **		U = 80 *		U = 66 NS		NA	
21) Conciliatory tendency up the hierarchy (Tau-Kr)	U = 91 **		U = 99 ***		U = 86 **		U = 92 ***		NA	
22) Conciliatory tendency and proximity of the partner (Tau-Kr)	U = 91 **		U = 97 ***		U = 89 **		U = 68 NS		NA	
23) Conciliatory tendency with valuable partners (Tau-Kr)	U = 90 **		U = 97 ***		U = 84 **		U = 51 NS		NA	

1 Kendall rank correlations.

2 Tau-Kr correlations.

3 Mann Whitney- U test.

Two tailed p-values combined with the improved Bonferroni method: * p<0.05, **p<0.01, ***p<0.001.

**Table 7:** Comparison between different variables of the complete model and the model without fear of defeat at high intensity of aggression (Mann Whitney U test, two-tailed).

	Complete Model	No fear of defeat	Mann Whitney U test
1) Percentage of time spent grooming	13–23%	19–24%	U = 92 **
2) Percentage of interaction time spent grooming	45–59%	72–77%	U = 100 ***
3) Centrality	−0.56*	−0.41*	U = 76 *
4) Conciliatory tendency	7–17%	19–28%	U = 100 ***
5) Reconciliation with valuable partners	0.21**	0.04*	U = 92 ***

The percentage of time females spend in fighting (or in grooming) is calculated as the number of fights (grooming) in the group divided between the total number of activations. The percentage of interaction time spend in grooming is the frequency of grooming divided by that of grooming plus that of fighting.

The hierarchical differentiation among all females was measured by the coefficient of variation of *Dom* values (standard deviation divided by the mean). For each run the average value was calculated (over period 200–260) and this was averaged over 10 runs. Higher values indicate greater rank distances among individuals [41]. Hierarchical differentiation is also reflected in the empirical behavioural measure of the degree of unidirectionality of aggression [12],[93], which we show also (Table 4).

The diversity of partners with whom a female interacts is measured by the Berger-Parker dominance index [94] by dividing an individual's frequency of grooming with its most favourite partner by its total grooming frequency.

The rank of group members we calculated as the average *Dom* value of each individual per run over periods 200–260. We used an average measure, because we correlated it with an average measure of aggressive and affiliative acts, i.e. data were summed over the whole interval of period 200–260. Apart from the average dominance value as a measure of rank we applied also a behavioural measure used in empirical studies, namely the average percentage of winning [95].

The degree to which dominant individuals (both males and females) occupy the centre was measured by a correlation between an individual's average *Dom* value and the average spatial direction of others around it. The centrality of each individual is calculated by means of circular statistics by drawing a unit circle around ego and projecting the direction of other group members as points on the circumference of this circle [96]. The connection of these points with ego's location results in vectors. The length of the mean vector represents the degree to which group members relative to ego form a cluster. Thus, longer mean vectors indicate a more peripheral, and hence, less central location of ego. Therefore, centrality of dominants is represented by a negative correlation between rank and the length of average vector (indicating the average direction of others).

In empirical studies reconciliation has been measured often by the PC-MC method (i.e. Post-Conflict versus Matched-Control). Here, we have used its improved version [16],[97]. In it a comparison is made between the moment in which grooming occurs during a short interval after a conflict, the so-called Post-Conflict period, and the moment it occurs in a control period of the same length (ten minutes), the Matched-Control period, taken a day later at the same time. Because our model is event-driven, we use its average number of fights over the interval 200–260 (of 2196 acts at high intensity) and the average frequency of fights per hour of rhesus monkeys of 0.2 per hour per individual [98] and a day length of about 13h to estimate that the interval of ten minutes is approximated by three activations in the model (one activation is about 3.8 min) and the interval of one day is approximately represented by one period. These are of course abstractions but results appear to be robust (see Sensitivity to parameter changes). Dyads were classified as ‘attracted’, when grooming happened earlier in the Post-Conflict period than in the Matched-Control. Pairs were classified as ‘dispersed’, when grooming happened in the reverse way, and as ‘neutral’, when grooming happened exactly at the same time or did not happen at all. Following [97], we calculated the conciliatory tendency, CT, of the group as:
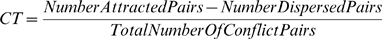
To measure the conciliatory tendency of each female with each of its group members, we calculated per pair the number of times they groomed sooner after a fight than in the matched control (attracted events) minus the cases where the opposite happened (dispersed events) divided by the total number of fights of the pair.

Correlations between the distribution of grooming, proximity, aggression and reconciliation among females, and between social interactions and rank were measured by means of the Tau-Kr correlation as described by Hemelrijk [93],[99], which is frequently used in studies of animal behaviour [100]–[102]. The advantage of this statistic is that it is animal-centred, because it takes variation in grooming and aggression between individuals into account by measuring the correlation between the corresponding rows of two social interaction matrices and because it accounts for the dependence of data in an interaction matrix. The level of significance was calculated using 2000 permutations [93],[99]. We tested for unidirectionality of attack and reciprocity of grooming by correlating an actor and receiver matrix with the Tau-Kr correlation. Note that unidirectionality and reciprocity are opposite correlations: a significantly negative correlation implies unidirectionality, whereas a significantly positive correlation implies reciprocity [93].

Whether grooming was directed up the hierarchy and to partners of similar rank was computed by the Tau-Kr correlation between, on the one hand, the grooming matrix and, on the other hand, respectively, the partner-rank-matrix (with the average *Dom* values of grooming partners in the rows) and the similar-rank-matrix (filled with zeros apart from the partners closest and second closest in rank, which are indicated as 1's). Note that the higher-ranking individuals have higher *Dom* values. Thus, a significantly positive correlation with the partner-rank-matrix corresponds to grooming being directed up the hierarchy, and a significantly positive correlation with the similar-rank-matrix corresponds to a high degree of grooming among individuals of similar rank.

To test the valuable-relationship hypothesis, we defined valuable relationships on the basis of the grooming frequency as is done by primatologists [e.g. 20],[21],[e.g. 23]. We used the frequency of grooming that occurred per dyad outside of the context of reconciliation in order for correlations with reconciliation not to be circular. We determined the degree of reconciliation with valuable partners by means of the Tau-Kr correlation between the matrices of the conciliatory tendency per dyad and that of the frequency of grooming per dyad outside the context of fighting (by subtracting the acts of conciliatory grooming from the matrix with all grooming acts). A significant positive correlation reflects that reconciliation is more frequent with partners that are more valuable.

## Results

### Empirical patterns

As regards our distinction of macaques in egalitarian and despotic, we updated the classification of Thierry [103],[104] with new data on Tibetan macaques (*Macaca thibetana*) [51] and Assamese macaques (*Macaca assamensis*) [105]. Therefore, we rated as egalitarian Barbary macaques (*Macaca sylvanus*), bonnet macaques (*Macaca radiata*), stumptailed macaques (*Macaca arctoides*), lion-tailed macaque (*Macaca silenus*), Celebes crested macaque (*Macaca nigra*) and tonkean macaques (*Macaca tonkeana*) and as despotic we classified long-tailed macaques (*Macaca fascicularis*), Japanese macaques (*Macaca fuscata*), rhesus macaques (*Macaca mulatta*), pigtailed macaques (*M. nemestrina*), Tibetan macaques and Assamese macaques. Regarding the dominance style (Table 1), the frequency of unidirectional aggression, which is a measurement related to the hierarchical gradient in macaques, appears to be higher in despotic than in egalitarian species [12]; further, frequency of aggression is lower [103] and average distance among all females is greater [103],[106]. Besides, for the despotic Japanese macaques, it has been reported that dominants are in the center of the group [107]–[109]. As to affiliative patterns, reconciliation occurs in both types of species, and is more frequent in egalitarian species [12]. Grooming is reciprocated in both egalitarian and despotic species. Further, grooming is directed up the hierarchy and to others of similar rank only in despotic species. Reconciliation is directed significantly more often to partners that are more valuable in several despotic species and according to a single study also in an egalitarian species, *Macaca arctoides* [19].

### Tuning the model

As described in the methods, we first tuned the percentage of time spent on grooming at a high intensity of aggression so that it resembled that of empirical data for despotic macaques [90]. Subsequently, we, unexpectedly, observed reconciliation. Since there are more precise data on the conciliatory tendency of despotic macaques than on their percentage of time spent on grooming, we subsequently tuned the conciliatory tendency to that of despotic macaques by adjusting the risk sensitivity further (7 in Table 4).

### Emergent patterns of dominance style and affiliation in the model

As to the two dominance styles in our model, we first confirmed that they still emerged, like they did in the earlier DomWorld model without grooming [41],[43]. In GrooFiWorld, at a high intensity of aggression, the hierarchy appeared to be significantly steeper than at a low intensity, aggression was more unidirectional, time spent on fighting was less, average distance among all females was greater and the spatial structure with dominants in the centre and subordinates at the periphery was more conspicuous (1–5 in Table 4; 1, 5–7 in Table 2C).

We confirm the resemblance of the affiliative patterns in the model to empirical data (Tables 1, 4): The conciliatory tendency appeared to be significantly higher at a low aggression intensity than at a high one (7 in Table 4); grooming appeared to be reciprocated at both intensities (8 in Table 4); a number of significant correlations were confined to a high aggression intensity, namely individuals direct their grooming significantly 1) up the hierarchy, 2) to others of similar rank, and 3) they reconcile more often with more valuable (grooming) partners (9–11 in Table 4). The only difference to empirical data concerns the absence in the model of more frequent reconciliation with valuable partners at low aggression intensity (11 in Table 4). However, in empirical data this correlation for the valuable relationship hypothesis was found only in a single empirical study of an egalitarian species [19] and it was based on a different method, i.e. the time rule method, whereas in the model we use the MC-PC method.

### Causation of patterns in the model and model-based hypotheses

In order to understand what caused these patterns of affiliation in the model, we took out four different assumptions in turn (see Parameters and Experimental Set-up). This reduced the number of emergent patterns. Switching off the self-reinforcing effect of the outcome of a fight did not affect the patterns qualitatively, but switching off the grooming-inducing effect of *Anxiety* changed three patterns of the 28 (11%) (indicated in bold in Table 5). Making grooming independent of fear of defeat changed seven patterns (29%) and choosing partners at random independent of spatial proximity changed 20 patterns (75%). Thus patterns arose mainly from the social-spatial group structure and secondly from grooming being dependent on fear of defeat.

To explain the emergence of each of the affiliative patterns in the model (Table 4), we proceed now by studying the effects of each of the four above-mentioned assumptions by taking them out (Table 5). This process leads to a number of model-based hypotheses for empirical data (Table 2).

The emergence of grooming up the hierarchy depended on grooming being based on fear of being defeated (without this assumption the pattern disappeared) and on the intensity of aggression (since it is absent at a low intensity of attack). This arises because the hierarchical differentiation is stronger at a high aggression intensity compared to a low one, and aggression is more unidirectional (1, 2 in Tables 4 and 5). Thus, when grooming depends on fear of defeat and the difference in rank between the partners is high, lower ranking ones will usually groom higher ranking ones and rarely attack them (as a consequence of Eq 1).

Grooming reciprocation (8 in Table 4, 5) arose from spatial structure, because it was disrupted only by taking out the socio-spatial structure, but not by taking out any of the other three assumptions. This means that, because certain individuals are often in close proximity, they will groom each other mutually, resulting in reciprocation. Furthermore, reciprocation appeared to be strongest in the experimental control condition where grooming did not depend on fear of defeat, and next strongest at a low aggression intensity. This arose because reciprocation was weakened less by differences in dominance, because these are smaller at low intensity of aggression (1, 2 in Table 4 and 5).

Besides, at high aggression intensity, but not at a low one, individuals automatically more often groomed partners that were similar in rank. This was due to grooming being based on fear of defeat, and due to spatial structure (10 in Tables 4 and 5). At a high intensity of aggression, not only a steep hierarchy develops, but also a spatial structure with dominants in the centre and subordinates at the periphery that is clearer than at a low intensity (1, 5 in Table 4; 1, 7 in Table 2C). Therefore individuals of similar rank are closer together. Thus, at high aggression intensity because individuals will groom up the hierarchy, while meeting mostly others of similar rank, this means that everyone grooms those of similar rank more often than those of lower rank, and, those of similar rank approximately as often as those of higher rank. Therefore, a correlation for grooming among individuals of similar rank results. At a low intensity of aggression, spatial centrality of dominants is absent (5 in Table 4) and due to the small rank differences grooming is directed neither up the hierarchy, nor to others of similar rank (9, 10 in Table 4).

The occurrence of reconciliation in our model is a side-effect of spatial proximity, since it is almost absent if interaction partners are chosen at random (7 in Table 5). Thus, reconciliation in the model is largely due to the higher probability of two opponents to be close to each other immediately after a fight (i.e. Post-Conflict) than otherwise (during the Matched-Control).

Furthermore, the conciliatory tendency is reduced at high intensity of aggression as a side-effect of the spatial structure and the dependence of grooming on the fear of defeat; without these assumptions the conciliatory tendency is independent of intensity of attack (7 in Table 5). This happens for three reasons (to be tested in empirical data, Table 2): at a high aggression intensity the spatial structure is more static (10 in Table 2C), average inter-individual distance is larger (6 in Table 2C), and centrality of dominants is greater (7 in Table 2C). First, spatial structure is more static at high aggression intensity, which is apparent from the stronger spatial assortment by rank of individuals (5 in Table 4), from the lower diversity of partners at high intensity of aggression than at a low one (16 in Table 6, 10 in Table 2C), and from the fact that the correlation between proximity and conciliatory tendency is significantly stronger at a high aggression intensity than at a low one (22 in Table 6; 11 in Table 2C). Therefore, former opponents may have been more often close to each other not only immediately after the conflict (in the post conflict period) but also in the matched control. Consequently, it is more likely that they groom each other in the matched control. If this happened at an earlier moment than after the conflict (in the post conflict period) it reduced the conciliator tendency. Second, due to the greater differences in rank, individuals are further apart (1, 4 in Tables 4 and 5) and groom less often both as calculated as the percentage of time and the percentage of interactions at a high than at a low intensity of aggression (13, 14 in Table 6; 8, 9 in Table 2C). Thus, they will automatically also groom less often immediately after a conflict, thus reconcile less than at a low aggression intensity (7 in Tables 4, 5). Third, at a high intensity of aggression grooming and conciliatory tendency are reduced because of the combination of spatial structure and the fear of defeat: If the fear of defeat is removed, the conciliatory tendency at a high intensity of aggression is higher than in the complete model (4 in Table 7), because spatial assortment according to dominance (i.e. spatial centrality of dominants) is weaker than in the complete model (3 in Table 7). Thus, dominants are relatively less often activated (to fight) and this increases the relative frequency of grooming because subordinates are aggressive less often (2 in Table 6). Thus without fear of defeat the percentage of time spent and interaction time spent on grooming is higher (13,14 in Table 6), so that it is higher than it is at a high intensity of aggression in the complete model (1, 2 in Table 7) and thus the percentage of time spent on reconciliation is higher also (5, 6 in Table 2A). Similarly, in the complete model, because at a lower intensity of aggression spatial structure is weaker than at a high intensity also, the percentage of grooming of the total number of interactions (aggressive plus grooming) is higher at a low than high intensity of aggression (14 in Table 6). Thus the conciliatory tendency is lower at a high than low intensity also (5, 6 in Table 2A).

Further, at a high intensity of attack reconciliation was directed mostly to those partners that are more valuable (in terms of grooming outside the context of reconciliation, 11 in Table 4) and this was stronger than at low intensity (23 in Table 6). This is due to (1) stronger effects of spatial proximity (2) high intensity of attack, and (3) fear of defeat, because without these traits there is no reconciliation with valuable partners (11 in Table 5) or it is significantly weakened (23 in Table 6). As regards spatial proximity, the stronger correlation for valuable relationships arises because the spatial structure at a high intensity is more rigid and therefore both reconciliation and grooming are correlated stronger with the proximity between partners than at a low intensity (20 and 22 in Table 6; 11 in Table 2C); thus, the two patterns of grooming and reconciliation are correlated too at a high, but not at a low intensity (11 in Table 4). As to the second and third cause, at a high intensity of aggression (due to the strong hierarchical differentiation) conciliatory tendency like grooming behaviour appears to be directed up the hierarchy (9 in Table 6; 1 in Table 2B), although this holds only when grooming is done out of fear of defeat (9 in Table 6) like in ‘normal’ grooming which does not occur after a conflict (9 in Table 4, 5).

### Other patterns in the model and model-based hypotheses

There are other patterns in the model that are of interest in it self and for study in empirical data (Table 2). For instance, in the model higher ranking individuals appear more aggressive due to the lower risk involved (2 in Table 6), and less anxious (but only at high intensity of aggression) (5 in Table 6; 12 in Table 2C) because they have lost fewer fights (4 in Table 6) and these effects are stronger at a high than low aggression intensity (10–12 in Table 6; 4 in Table 2C).

Further, both at a high and a low intensity of aggression, there is no correlation between grooming and rank (6 in Table 6; 2 in Table 2A). This is remarkable at high intensity of aggression, because lower ranking individuals are more anxious and therefore, they may be expected to groom others more often. The absence of this correlation arises from the fact that a high grooming frequency by low ranking individuals is counteracted by the spatial social structure (5 in Table 5); due to their peripheral positions, low ranking individuals have fewer opportunities to interact with others than dominants do and therefore, despite their greater tendency to groom, they do not groom more often than dominants.

### Sensitivity of patterns to parameter changes and the measure of rank

As regards the sensitivity to changes of parameter, the affiliative patterns were insensitive to different values of parameters related to *Anxiety*. Values ranging from 0.001% to 10% for *AnxInc* and values from 0.05 to 0.15 for *AnxIncFight*, *AnxDcrGree* and *AnxDcrGrmr* (whereby *AnxDcrGree* was kept at higher values than *AnxDcrGrmr*) changed the level of anxiety, but did not change results qualitatively.

To obtain a sufficiently high number of interactions (both of grooming and fighting) to detect affiliative patterns statistically, a *Perspace* 8 was necessary, whereas a value of 4 was too low. Furthermore, two mental fights (Equation 1) before initiating a dominance interaction (*RiskSens* = 2) were needed in order to make the frequency of grooming higher than that of fighting like in empirical data. Besides, in empirical data the percentage of time spend fighting was lower in fierce than mildly aggressive species. This was true when comparing the percentage of fighting at high versus low intensity of aggression in the model for *RiskSens* 1 and 2, but not for higher values of *RiskSens*.

Results of reconciliation were similar if we prolonged the period of Matched control from three activations to five and to ten activations (Puga-Gonzalez *et al* in prep).

Since in the empirical data average dominance cannot be accessed directly like in our model, we also tested all correlations with a measure of dominance, i.e. their average percentage of winning, which can be measured in real behaviour [95]. All results of Table 4 and 6 remain similar (also in the strength of the significance), apart from two correlations in Table 6: when correlating with the average percentage of winning as a measure of dominance, at a high intensity of aggression, higher ranking individuals groom others significantly less and at a low intensity of aggression, the negative correlation between aggression received and dominance is no longer significant (data available on request). It should be noted however, that to explain patterns of our simulation, the correlations with average dominance value are of greater interest than with percentage of fight won because the average dominance value is a more direct cause of behaviour in the model.

## Discussion

Our model presents us with an integrative theory of affiliative behaviour in primates, because it gives a coherent explanation for aspects of many of the patterns of affiliation typical of egalitarian and despotic macaques. It does so, while it only makes the ‘cognitive’ assumptions that individuals are 1) intending to group, 2) they recognise each others rank (here it is unspecified whether this is due to the other's body posture, former experiences with the other or due to observations of interactions among other group members, or some or all of these), 3) in their initiation of aggression they are sensitive to risks of losing a fight, 4) their grooming is induced by the expectation of losing a fight, and 5) the wish to decrease their anxiety. Anxiety is induced by fighting and increases with the duration of not being groomed. Thus, remarkably, in contrast to views of others [4],[6],[13],[15], our model ignores a number of the specific cognitive assumptions that have been made for primates. In it individuals only need minimal information. Thus, our model generates a) reciprocation without that the individual keeps records in its memory of services given to and received from each of its partners, b) grooming up the hierarchy without an intention to receive support in exchange, c) grooming others of similar rank without competition for higher-ranking grooming partners or attraction to higher ranking-partners, d) reconciliation without a conciliatory predisposition or a memory of, and a selective attraction to, a former opponent and e) reconciliation with partners that are more valuable without any estimate of the quality of the relationship. Besides, it reproduces the differences between egalitarian and despotic species in their conciliatory tendency without a difference between low and high intensity of aggression in possibilities to negotiate [110] and without reconciliation reducing conflict escalation [111]. Our model also provides us with coherent mechanisms for the systematic variation hypothesis or the co-variation hypothesis [12], [18], [75], [103], [104], [112]–[114].

As to the function of reconciliation, our model does not represent this specifically in its rules, since reconciliation emerges from a rule that makes individuals groom merely to reduce anxiety. Thus, its function is to reduce anxiety. However, in our model (like in reality) grooming occurs more often after a fight than at other times. Therefore, if similar processes in reality cause patterns of reconciliation, such emergent patterns of reconciliation after a fight may still function to repair a relationship.

### Causation of patterns in the model

The causation of each of the affiliative patterns in the model is as follows. First, grooming up the hierarchy results when aggression intensity is high and the hierarchy is steep because individuals seldom dare to attack higher ranking ones, and therefore in order to reduce their anxiety they groom up the hierarchy instead. When aggression intensity is low, the hierarchy is weak, thus individuals experience a smaller risk to attack higher ranking ones and therefore there is no such pattern.

Other patterns depend on the spatial configuration. Because interactions take place in space, individuals are more likely to be close to those they have recently interacted with than to others. Therefore, they are more likely to groom one another after an interaction than at other times. Thus, we observe patterns of both reconciliation of fights and reciprocation of grooming. In the model, aggression determines the spatial structure of the group [43]. At a high intensity of aggression a spatial structure develops through the continuous fleeing of low ranking individuals. Therefore, subordinates end up at the periphery and dominants are located in the centre, and thus individuals are closer to others of similar rank. Such a rank-assortment is virtually absent at a low intensity of aggression [41]. Therefore at a high intensity of aggression, since individuals are closer to others of similar rank, they usually groom others of similar rank. Furthermore, at a high intensity of aggression dominants interact more often than subordinates, because dominants are surrounded at all sides by others due to their spatial centrality. Consequently, because dominants are more often aggressive than subordinates are, the percentage of interaction time spent in grooming is lower at a high than at a low intensity of aggression. Because individuals groom relatively less often, this causes less reconciliation at a high than at a low aggression intensity. Furthermore, due to the fact that the spatial structure is relatively more rigid at a high aggression intensity, individuals are more often close to the same partner and this increases the chance that they are close to a former opponent at all times. Therefore, the frequency with which individuals groom with former opponents sooner after a fight than in the matched control (MC-PC method) declines. This reduces the rate of reported reconciliation. Besides, due to the relatively rigid spatial structure at a high aggression intensity, individuals more often reconcile with the same partners as they groom with and thus, they reconcile with valuable partners more often than at a low aggression intensity. In sum, aggression structures the spatial configuration of individuals in the group and (together with grooming out of fear of defeat) this structures the affiliative patterns.

### Relevance to empirical data

Although in empirical data rank is not measured by an internal Dom value (like in our model), similar results were obtained in the model if rank was computed by the empirical measure, the average percentage of winning [95]. In the model, the correlations with rank and 1) aggression given, 2) aggression received and 3) fights lost appeared to be stronger at a high intensity of aggression than at a low one (10–12 in Table 6). Whether this difference may serve as a new indication of the degree of despotism for real primates, needs further study (2–4 Table 2C).

The relevance of the model to affiliative patterns of primates is supported by the following empirical evidence (Table 2). First, in many species grooming up the hierarchy appears to be stronger the steeper the gradient of the hierarchy when comparing between groups of a single species [115] (conform 3 in Table 2A). Further, the larger inter-individual distance at high versus low aggression intensity in the model (6 in Table 2C) is confirmed in empirical data at several levels of comparison, not only by a comparison between species, namely between rhesus and tonkean macaques [103], and between rhesus and stump-tailed macaques [106] (see Table 1), but also within groups intense conflicts result in larger distances between opponents than do mild conflicts in both a group of Japanese macaques [116] and wild chimpanzees [117]. The correlation between proximity and grooming (1 in Table 2A) is supported in lion-tailed macaques and tonkean macaques [52],[118] and by the difference in distance and grooming frequency between despotic rhesus monkeys and egalitarian stump-tailed macaques [106]. The combination of spatial configuration and proximity induced grooming leads to reciprocity of grooming. This mechanism may underlie the so-called ‘symmetry-based’ reciprocity [119] where the correlation results from a common underlying variable, namely proximity.

As to the extent to which closer proximity between former opponents after a fight explains the occurrence of the higher grooming tendency after a fight (which is interpreted as reconciliation), a number of empirical studies confirm this. These studies concerned stump-tailed macaques, rhesus macaques [19],[120], Japanese macaques [116], Moor macaques [121] and a comparison between studies of several species in captivity vs. natural conditions [122].

However, a number of studies conclude that closer distance after a fight cannot explain the conciliatory tendency exhaustively, because when controlling for distance by matching (to some degree) the distance in the matched control to that after the fight (in the post conflict period), these studies show that a certain conciliatory tendency still remains after controlling for distance [21], [63], [123]–[126] despite a great reduction in the conciliatory tendency in some studies [120]. Whether this also happens in the model if we control for distance in the matched control of the MC-PC method, we will study in future.

Further, as in the model (21 in Table 6; 1 in Table 2B), females of a group of baboons reconciled more often with higher ranking victims than lower ranking ones [127]. In the model, this arises at a high aggression intensity, because individuals groom others of higher rank more often, since they are afraid to attack them. Thus, they also groom high ranking ones after a fight more often and thus reconcile with others of higher rank more. Note that this finding also may be interpreted in the frame of the most valuable relationship hypothesis, because the higher the rank of the partner (due to the effective support it can give, for instance) the more valuable the individual is to reconcile with.

Further, as in the model, a correlation between rank and grooming is lacking (2 in Table 2A) in the study of baboons and vervets (which are despotic species) [128],[129], but such a correlation is found in lion tailed macaques (in this study this species appears to be despotic) [52]. Since the absence of this correlation in the model is due to spatial centrality of dominants, we expect spatial social structure to be stronger in baboons and vervets than in lion tailed macaques.

At a high intensity of attack, but not at a low one, lower ranking females are more anxious (5 in Table 6; 12 in Table 2C), because they more often receive aggression and lose fights than higher ranking individuals in the model (10–12 in Table 6; 3–4 in Table 2C). This is confirmed by correlations between the frequency of receipt of aggression, the level of anxiety, and anxiety-induced arthrosclerosis in the fiercely aggressive despotic macaque species, rhesus and long-tailed macaques [130]–[132]. It is of interest to see whether in empirical data, like in the model (5 in Table 6) this correlation between rank and anxiety is weaker in egalitarian species (12 in Table 2C).

Thinking along the lines of dominance relations, our model may also change our explanations for two other phenomena. Firstly, in female-bonded species, in primate groups that are more female-biased females appear to groom less frequently. This is explained by the assumption that in female-bonded groups not every female needs to groom every other [133]. According to our model, however, reduced grooming by females in a group that is female-biased may be a side-effect of the rule that individuals groom the others out of fear of defeat: Because in a female-biased group females meet other females more often and they fear defeat less if they meet a female than if they meet a male, they will attack more than in a group with more males. Second, the fact that female macaques groom males more often than vice versa [118], [134]–[137] is explained by our model as a consequence of their subordinance to males. From this we may derive another prediction: since in despotic species females are dominant over a higher number of males than in egalitarian species [45], we expect that (for the same adult sex ratio in despotic and egalitarian groups) females of despotic species groom males less than females of egalitarian species do (13 in Table 2C).

### Shortcomings and benefits of our model

Our model shows the four different levels of complexity of social behaviour distinguished by Hinde [138]: Individual behaviour, interactions, relationships and social structure. In agreement with Hinde'suggestion, each level can be described in terms of the level below it, and levels influence each other mutually. For instance, the nature of the behaviour of the participants influences their relationship and these relationships in turn, also influence the behaviour of the participants. Also related to this view is that observed social structure can vary dramatically with circumstances, without any changes in the underlying motivational mechanisms or strategies. For instance, here we show that patterns of reconciliation differ depending on intensity of aggression and in our former paper we showed that female dominance increases with the percentage of males in the group [45].

A criticism made against DomWorld by Bryson and co-authors [139] has been that the dominance hierarchy in the model was not as stable as that of real primates. The dominance hierarchy in GrooFiWorld is stable, however, because average dominance values between periods 200 and 230 are significantly correlated with those between 231 and 260 (see methods). Further, in GrooFiWorld we have shown that even if we keep the hierarchy 100% stable (by omitting the self-reinforcing effects of winning and losing fights) all patterns remain similar (Table 4, 5).

Another criticism concerned the directional inconsistency of aggression [140]. The directional inconsistency of aggression at high aggression intensity in DomWorld appeared to be lower than that in empirical data. In the present paper, in GrooFiWorld, the directional inconsistency is higher than in DomWorld. 0.73 vs 0.55 respectively, because in GrooFiWorld the individuals think twice before they attack, whereas in DomWorld they think only once and thus, attack higher ranking individuals more often. How it compares exactly to empirical data is not clear, because the matrices tested by de Vries sometimes comprise of males, sometimes of females and sometimes of both sexes and the directional inconsistency probably depends on the group composition. However, despotic macaque species show an average directional inconsistency of 0.89, which still is above that of GrooFiWorld. To study this in more detail is beyond the scope of this paper.

Yet, there are other shortcomings in our study of the model that will be amended in future. There are a number of patterns related to reconciliation that have been found in studies of real primates that we do not yet treat in the model [25], [110], [127], [141]–[143], we used the time rule method [58] neither to test for reconciliation nor for the valuable relationship hypothesis, nor did we control for proximity in our study of reconciliation [63]. The rule of grooming out of fear of defeat may be interpreted by assuming that individuals groom others to calm these partners down and to forestall the chance of receiving aggression from them; thus, it could be viewed as an exchange of grooming for tolerance. However, in the present model grooming others does not influence whether or not the groomee will subsequently attack the groomer. The model also does not represent cases in which grooming can be rejected by the receiver, nor pre-existing differences between individuals, such as are apparent, for instance, between primates of different personality [144], nor what individuals compete for such as sex or food. It omits kin-relations and offspring among partners as well as coalitions. Besides, we have not yet studied effects of different sex ratios, whereas primate groups of the same species may differ in sex ratios, and this has been shown to have an influence on their affiliative patterns [133], [145]–[147]. These are natural variations and extensions that will need to be added to our model, as we intend to do in future studies.

As to cognition, our model does not at all reflect the behavioural and cognitive complexity of primates. Regarding affiliation, it is confined only to the representation of an anxiety reducing effect of grooming in the context of a competitive regime. Because of the resemblance of the emergent affiliative patterns in our model to those of primates, similar processes may cause these affiliative patterns in primates also. Whether or not primates may (sometimes) use the more complex cognitive rules that have been suggested by primatologists before, our model cannot decide. Instead, our model may be used as a null-model that indicates what patterns we should expect in the absence of the usual cognitive rules regarding reciprocation, reconciliation etcetera. Thus, it does not deny that primates are intelligent as has been shown in many experimental studies [37],[38],[148], but it questions whether these primates use all aspects of their intelligence in all contexts. It illustrates that apart from the here reproduced patterns at a group level in the model, extra evidence, is needed as proof of 1) intentional reciprocation, 2) competition for higher ranking grooming partners, and 3) intentional exchange and 4) intentional reconciliation. Further, our model points to the need for more studies of the spatial distribution of monkeys within a group. Of these studies [108],[149] (Girod, Thierry, Hemelrijk, in prep), there have been only a few so far.

In sum, we have shown that without the specific cognitive assumptions for the creation of each pattern of grooming, cognitively simple local interactions and self-organization suffice to produce many of the affiliative patterns that are typical of egalitarian and despotic primate societies (Table 1, 4) and also a number of other patterns (Table 6). The main finding is that the spatial configuration associated with the competitive regime and grooming out of fear of defeat or out of anxiety structure the patterns of grooming such that we measure patterns of reciprocation, exchange and reconciliation. This leads to a number of model-based hypotheses for real primates (Table 2). Because the model generates many of the behavioural patterns found in real primates, but does so without the usually assumed cognitive processes, it can be used as a null model for studying primate affiliative behaviour.

## Supporting Information

Video S1 DemoGrooFiWorld. Individuals are represented by circles, their headings are given by a red arrow and their activity is indicated by their colour. If it moves, turns or rests, it is light blue; if it fights, it becomes dark blue; and if it grooms, it turns green. Note that after grooming individuals turn away from each other. Several cases of reconciliation can be observed, for instance: From second 7 to 10: two individuals on the upper left side of the screen fight at around second 7 and they reconcile at around second 10. From second 36 to 39: two individuals on the left side of the screen fight at around second 36 and they reconcile at second 39. From second 31 till 49: Two individuals on the upper right side of the screen fight 3 times from second 31 to 38 and they reconcile subsequently around second 49.
(1.34 MB AVI)
